# Physiological Effects and Inter-Individual Variability to 12 Weeks of High Intensity-Interval Training and Dietary Energy Restriction in Overweight/Obese Adult Women

**DOI:** 10.3389/fphys.2021.713016

**Published:** 2021-07-29

**Authors:** Omar Andrade-Mayorga, Nicolás Martínez-Maturana, Luis A. Salazar, Erik Díaz

**Affiliations:** ^1^Center of Molecular Biology and Pharmacogenetics, Scientific and Technological Bioresource Nucleus (BIOREN), Universidad de La Frontera, Temuco, Chile; ^2^Department of Preclinical Sciences, Faculty of Medicine, Universidad de La Frontera, Temuco, Chile; ^3^Exercise, Movement, and Health Research Group, Universidad de La Frontera, Temuco, Chile

**Keywords:** exercise, high-intensity interval training, overweight, interindividual variability, individual response, women

## Abstract

**Background**: Human adaptive response to exercise interventions is often described as group average and SD to represent the typical response for most individuals, but studies reporting individual responses to exercise show a wide range of responses.

**Objective**: To characterize the physiological effects and inter-individual variability on fat mass and other health-related and physical performance outcomes after 12 weeks of high-intensity interval training (HIIT) and dietary energy restriction in overweight/obese adult women.

**Methods**: Thirty untrained adult overweight and obese women (age = 27.4 ± 7.9 years; BMI = 29.9 ± 3.3 kg/m^2^) successfully completed a 12-week supervised HIIT program and an individually prescribed home hypocaloric diet (75% of daily energy requirements) throughout the whole intervention. High and low responders to the intervention were those individuals who were able to lose ≥ 10 and < 10% of initial absolute fat mass (i.e., kilograms), respectively.

**Results**: The prevalence for high and low responders was 33% (*n* = 11) and 66% (*n* = 19), respectively. At the whole group level, the intervention was effective to reduce the absolute fat mass (30.9 ± 7.2 vs. 28.5 ± 7.2 kg; *p* < 0.0001), body fat percentage (39.8 ± 4.3 vs. 37.8 ± 4.9%; *p* < 0.0001), and total body mass (76.7 ± 10.1 vs. 74.4 ± 9.9 kg; *p* < 0.0001). In addition, there were improvements in systolic blood pressure (SBP; Δ% = −5.1%), diastolic blood pressure (DBP; Δ% = −6.4%), absolute VO_2_peak (Δ% = +14.0%), relative VO_2_peak (Δ% = +13.8%), peak power output (PPO; Δ% = +19.8%), anaerobic threshold (AT; Δ% = +16.7%), maximal ventilation (VE; Δ% = +14.1%), and peak oxygen pulse (O_2_ pulse; Δ% = +10.4%). However, at the individual level, a wide range of effects were appreciated on all variables, and the magnitude of the fat mass changes did not correlate with baseline body mass or fat mass.

**Conclusion**: A 12-week supervised HIIT program added to a slight dietary energy restriction effectively improved fat mass, body mass, blood pressure, and cardiorespiratory fitness (CRF). However, a wide range of inter-individual variability was observed in the adaptative response to the intervention. Furthermore, subjects classified as low responders for fat mass reduction could be high responders (HiRes) in many other health-related and physical performance outcomes. Thus, the beneficial effects of exercise in obese and overweight women go further beyond the adaptive response to a single outcome variable such as fat mass or total body mass reduction.

## Introduction

Human adaptive response to exercise interventions is often described in general terms, assuming that the group average and SD are sufficient to represent the typical response for most individuals (Mann et al., 2014). However, studies reporting individual responses to exercise are usually heterogeneous, showing a wide range of responses to the interventions rather than a similar response (Bouchard and Rankinen, 2001; King et al., 2008; Scharhag-Rosenberger et al., 2012; Bonafiglia et al., 2016; Gurd et al., 2016; Parr et al., 2016; Álvarez et al., 2017a; de Lannoy et al., 2017; Sparks, 2017; Williamson et al., 2017; Chrzanowski-Smith et al., 2019; Ross et al., 2019). Thus, research attempting to quantify, predict, and explain inter-individual variability to exercise training has grown gradually (Chrzanowski-Smith et al., 2019). This heterogeneity has been linked to physiological, genetic, and epigenetic factors (Sparks, 2017; Hagstrom and Denham, 2018). Following this, recent literature has dichotomously classified individuals as either “responders/non-responders” or “high responders (HiRes)/low responders (LoRes)” using a pre-determined threshold. The most commonly used criteria are: clinical cut-off points (Mann et al., 2014; Parr et al., 2016; Álvarez et al., 2019), within-subjects coefficient of variation (CV) (Scharhag-Rosenberger et al., 2012; Astorino and Schubert, 2014), typical error of measurement (TE) (Ross et al., 2015; Montero and Lundby, 2017), or two times the typical error (2x TE) (Bouchard et al., 2012; Bonafiglia et al., 2016, 2018; Gurd et al., 2016; Raleigh et al., 2016; Álvarez et al., 2017a; de Lannoy et al., 2017; Astorino et al., 2018). Concerning exercise, most studies have focused on heterogeneity of the cardiorespiratory fitness (CRF) adaptations to training (Bouchard and Rankinen, 2001; Scharhag-Rosenberger et al., 2012; Ross et al., 2015, 2019; Williamson et al., 2017). Few studies explore the inter-individual variability in body composition and exercise-induced fat mass changes (King et al., 2008; Álvarez et al., 2017a; Andreato et al., 2019). This research topic is essential because overweight and obesity have emerged as the leading health concerns during the last decades (Heymsfield and Wadden, 2017), and it is expected that by 2025 global obesity prevalence will reach 18% in men and surpass 21% in women (NCD-RisC, 2016). The global combined prevalence of overweight and obesity [i.e., body mass index (BMI) ≥ 25 kg/m^2^] is currently estimated to be over 38% (Ng et al., 2014). Furthermore, obesity is associated with expanding white adipose tissue (adipocyte hypertrophy) and visceral adiposity, producing metabolic dysregulation resulting from the associated pro-inflammatory and insulin-resistant phenotype (Vazquez-Carrera, 2016; Hepler and Gupta, 2017). Thus, adiposity is a strong predictor of morbidity and mortality (Verheggen et al., 2016). Obesity is usually treated through behavioral changes involving exercise and nutrition (Petridou et al., 2019). Exercise represents an effective strategy to reduce body mass and improve health, being the lack of exercise one of the main factors associated with obesity (Booth et al., 2012). Moreover, high-intensity interval training (HIIT) has proven to be superior to traditional exercise programs in reducing fat mass and improving CRF in adults with obesity (Turk et al., 2017). This finding is important because some results support the idea that women have particular metabolic characteristics such as greater reliance on fat oxidation than men during submaximal exercise. However, they also indicate that this greater fat oxidation is reached at higher exercise intensities in women than men (Cheneviere et al., 2011). Therefore, the present study aimed to characterize the physiological effects and inter-individual variability on fat mass and other health-related and physical performance outcomes after 12 weeks of HIIT in overweight/obese adult women.

## Materials and Methods

### Study Design and Participants

A group of untrained adult overweight and obese women were studied, all referred by a physician to the supervised exercise program in our research center. Ethical approval for the study was provided by Scientific Ethics Committee at Universidad de La Frontera (Protocol N°112/16). The study was conducted according to the Helsinki Declaration. All volunteers received information about the protocol and provided written consent before the beginning of the study.

**Figure 1:**
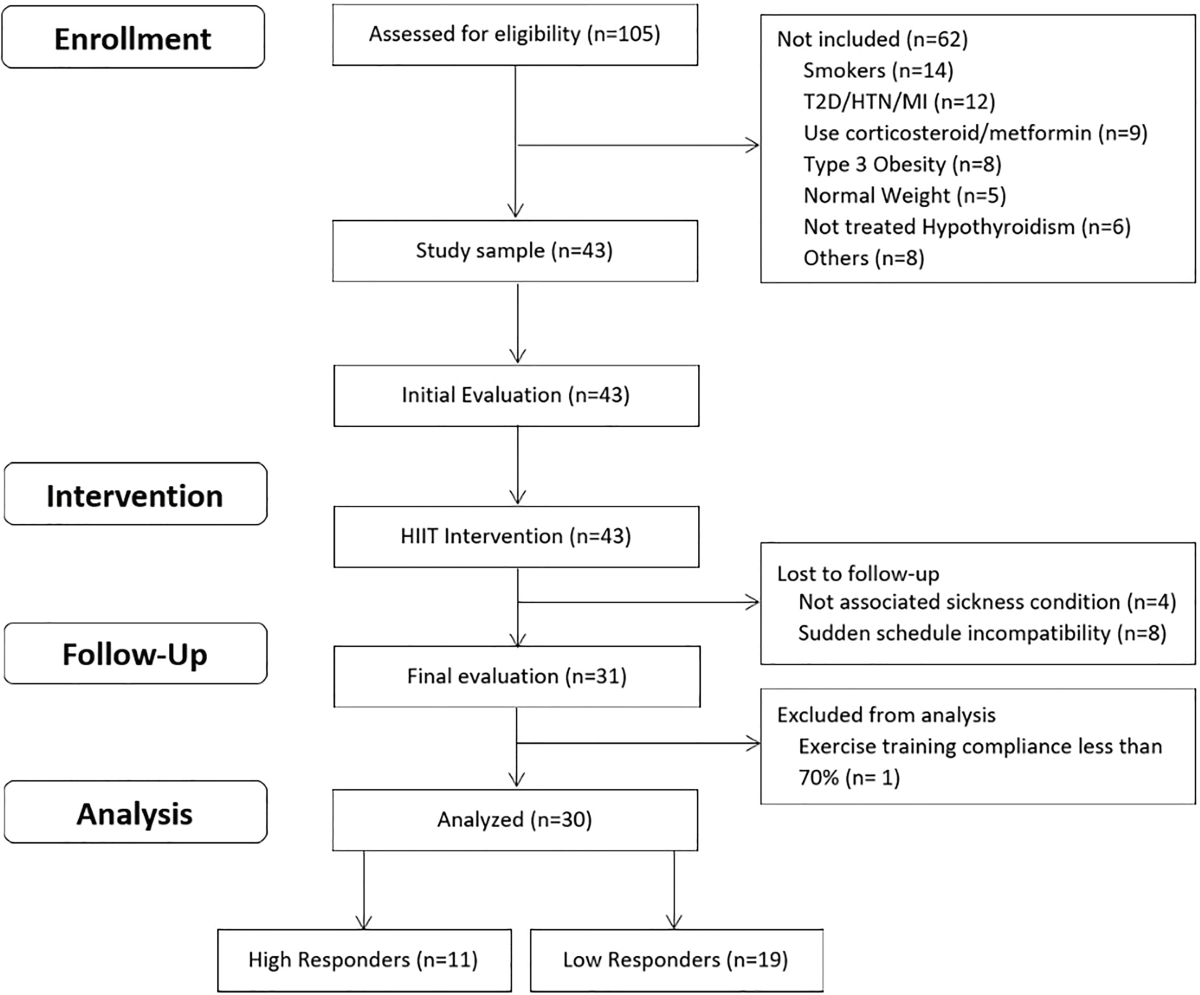
Study design.

The inclusion criteria were: (a) women aged 18–45 years; (b) diagnosed with overweight or type 1 or 2 obesity (BMI between 25 and 39.9 kg/m^2^); (c) untrained (not involved in regular physical activity or exercise program during the previous 6 months); (d) pre-menopausal women; and (e) previously screened by a physician professional. The exclusion criteria were: (a) previously diagnosed diseases, such as diabetes mellitus, hypertension, myocardial infarction, and class III obesity; (b) receiving pharmacologic corticoids, metformin, or other drugs that may affect metabolism; (c) smoking habit; (d) history of bariatric surgery; (e) untreated hypothyroidism; and (f) skeletal muscle disabilities or a specific indication to avoid exercise by medical reasons. Attendance to a minimum of 70% (26/36 sessions) of the exercise program duration was fixed as the cut-off point for individual compliance for subjects included in the final statistical analyses. A structured medical history record and physical examination were performed on 105 adult women for enrolment purposes. Forty-three subjects met all the inclusion criteria, and 30 of them were finally studied. This study used a non-probabilistic sample aiming to separate high and low responders to a 12-week exercise program. In this study, HiRes to exercise intervention were those individuals who were able to lose ≥ 10% of initial absolute fat mass (i.e., kilograms), and LoRes were individuals who lost < 10%, similar to previous studies that have also used clinical cut-off points for this classification (Milagro et al., 2013; Parr et al., 2016). The study design is shown in Figure 1. Anthropometry/body composition, endurance performance, resting blood pressure, fasting glucose and insulin, and lipids levels, were assessed before and after the 12-week follow-up. Participants performed a supervised progressive exercise program three times a week on non-consecutive days, and it consisted of high-intensity cycling intervals interspersed with recovery inactive periods. Dietary intake was considered a control variable. To this purpose, all subjects were assessed and had nutrition counseling by the study nutritionist and instructed to follow an individually prescribed home hypocaloric diet (75% of daily energy requirements) throughout the whole intervention.

### Anthropometric/Body Composition and Cardiovascular Measurements

The initial assessment was carried out to record socio-demographic, physical, and physiological characteristics. Body mass, fat mass, body fat percentage, and fat-free mass (FFM) were assessed with the subject barefoot, wearing underclothes, and no metal objects using a Tanita™ foot-foot bioelectrical impedance analyzer (BIA; Tanita Corporation, model BC-541, Japan). Its prediction formula has been previously validated, showing high reproducibility (Jebb et al., 2000). Height was measured without shoes to the nearest millimeter with a stadiometer (Seca model 213, Hamburg, Germany). BMI was calculated using the formula weight divided by height squared (kilograms per square meter). The systolic and diastolic blood pressures (DBPs) were determined using an automatic monitor (Omron HEM-7114; Omron Healthcare) in duplicate and after 15 min of complete rest with the subjects in a supine position. The heart rate (HR) was measured at rest in similar conditions using a telemetric heart rate sensor (Polar model V800, Finland).

### Endurance Performance Assessment/Incremental Exercise Test

Endurance performance was assessed 1 week before and after the 12-week intervention during an incremental exercise test designed to obtain peak oxygen consumption (VO_2_peak). In brief, the VO_2_peak test consisted of free-wheel pedaling for 2 min at 70–80 RPM on a cycle ergometer (Lode Corival, Groningen, The Netherlands), followed by an initial 50 W load for 2 min and 25 W increments every 2 min until the participant reached volitional fatigue. Gas exchange was collected throughout the test using an indirect calorimeter/ergospirometer system (Ultima CPX™ metabolic system, Medgraphics, Minnesota, United States), which was calibrated before the exercise test. Additionally, it was measured the peak power output (PPO), anaerobic threshold (AT), respiratory exchange ratio (RER), peak oxygen pulse (O_2_ pulse; VO_2_peak/HRmax during the exercise test), ventilation (VE), and respiratory rate (RR). HR was monitored with a continuous telemetric HR sensor (Polar model V800, Finland) throughout the whole test.

### Exercise Training Intervention

Participants performed a 12-week supervised HIIT program, with training sessions three times a week on non-consecutive days. The exercise session consisted of 1-min cycling at a high intensity (workload during each interval was set to achieve muscle failure at the end of 1-min exercise period and reaching ~85–100% maximal heart rate obtained during the incremental exercise test), followed by a 2-min inactive resting period (sitting on the cycle ergometer), and repeated 10 times (*1x2x10* protocol; 1:2:10 to work: rest: repetitions, respectively). In summary, the total duration of one session of the *1x2x10* protocol was 30 min, with 10 min of effective exercise training, and without a warm-up or cool-down period. All exercise sessions were individually supervised to achieve muscle fatigue at each exercise interval as the primary indicator of intensity together with a continuous heart rate monitoring (Polar V800, Polar™, Finland) in order to supervise that the chronotropic response was expected according to previously found with the same HIIT exercise protocol (Andrade-Mayorga et al., 2020). Load progression was defined as the gradual increase in workload developed when the subject failed to reach muscle failure at the end of the 60-s exercise interval, monitored individually on a series-by-series and session-by-session basis. Thus, the load progression increased in parallel to the increment in the work capacity of each individual.

### Dietary Assessment and Hypocaloric Diet

Participants were instructed to follow an individually designed hypocaloric diet [75% of estimated energy requirements (EERs)], equivalent to 1,354 ± 114.5 kcal/day, throughout the 12-week study period to control the dietary intake. A 24-h diet recall and a modified food choice questionnaire (7 days) were applied at baseline (Salvador Castell et al., 2015). Total energy expenditure (TEE) was estimated using the factorial method based on their reported daily physical activity (FAO/WHO/UNU, 2004; Levine, 2005). In brief, basal metabolic rate (BMR) was estimated using the Mifflin–St. Jeor equation (Cancello et al., 2018), additional caloric requirements were determined based on each subject’s physical activity level (PAL), which were used to calculate the PAL index, TEE, and the 75% EER. Subjects had individual monthly meetings with the nutritionist during the 3-month intervention to encourage compliance. Dietary compliance was assessed using an instrument adapted from the Perceived Self-Regulatory Success in Dieting Scale (Meule et al., 2012), where each subject’s perceived adherence was quantified using a five-point Likert scale. The nutritional intervention excluded the use of nutritional supplements.

### Statistical Analysis

GraphPad Prisma statistical software 7.0 (San Diego, CA, United States) was used. The normal distribution of all the variables was tested using the D’Agostino-Pearson test. All the continuous variables were expressed as mean ± SD. The differences between quantitative variables were analyzed by paired *t*-test (intra-group differences pre- and post-intervention) or unpaired *t*-test for independent groups (between-groups difference within time points). Gardner-Altman estimation plots, which show individual values, means, and effect size with a 95% CI, were developed using Estimation Statistics for Data Visualization (Ho et al., 2019). The correlation between the magnitude of the fat mass responses and baseline anthropometric measures was calculated using Pearson correlation coefficient with 95% CIs. The level of significance used in all comparisons was *p* < 0.05.

## Results

From the initial cohort, 30 adult women (age = 27.4 ± 7.9 years; BMI = 29.9 ± 3.3 kg/m^2^) successfully completed the 12-week intervention. All participants tolerated the exercise program well, and there were no injuries during the intervention. The baseline demographic, physical, and physiological characteristics of the participants are presented in Table 1.

**Table 1:** Baseline demographic, physical, and physiological characteristics of the participants.

Variable	All (*N* = 30)	Low responders (*N* = 19)	High responders (*N* = 11)	*p* value (LoRes vs. HiRes)
*Demography*				
Age (years)	27.4 ± 7.9	27.6 ± 8.6	27.2 ± 7.2	0.898
*Physical Activity*				
Physical activity level index	1.39 ± 0.08	1.38 ± 0.08	1.41 ± 0.09	0.437
*Metabolic and Nutritional*				
BMR (kcal/day)	1,295 ± 54.3	1,283 ± 55.7	1,314 ± 48.1	0.143
TEE (kcal/day)	1805 ± 152.7	1,778 ± 159.9	1852 ± 133.4	0.140
75% EER (kcal/day)	1,354 ± 114.5	1,333 ± 119.9	1,389 ± 100.1	0.140
*Anthropometry/Body composition*				
Body mass (kg)	76.7 ± 10.1	77.2 ± 9.8	76.1 ± 11.1	0.788
BMI (kg/m^2^)	29.9 ± 3.3	30.6 ± 3.3	28.8 ± 3.0	0.159
Fat mass (kg)	30.9 ± 7.2	31.6 ± 6.9	29.8 ± 8.0	0.533
Body fat percent (%)	39.8 ± 4.3	40.5 ± 4.0	38.6 ± 4.9	0.246
FFM (kg)	45.9 ± 3.5	45.6 ± 3.5	46.3 ± 3.5	0.610
*Cardiorespiratory fitness*				
VO_2_peak (ml·min^−1^)	1,800± 249	1,750 ± 230	1,888 ± 266	0.146
VO_2_peak/FFM (ml·kg^−1^·min^−1^)	39.2 ± 3.9	38.3 ± 3.6	40.7 ± 3.9	0.106
*Cardiovascular*				
O_2_ pulse (ml/beat)	10.3 ± 1.5	10.2 ± 1.6	10.4 ± 1.2	0.683
Systolic BP (mmHg)	110.2 ± 8.7	110.9 ± 8.4	108.9 ± 9.4	0.575
Diastolic BP (mmHg)	73.6 ± 7.5	73.2 ± 8.7	74.2 ± 5.2	0.739
Mean BP (mmHg)	85.8 ± 7.2	85.7 ± 7.9	85.8 ± 6.2	0.996

Data are shown as mean and ± SD. LoRes, low responders; HiRes, high responders; BMR, basal metabolic rate; TEE, total energy expenditure; EER, estimated energy requirement; BMI, body mass index; FFM, fat-free mass; VO_2_peak, peak oxygen consumption; and BP, blood pressure.

**Figure 2:**
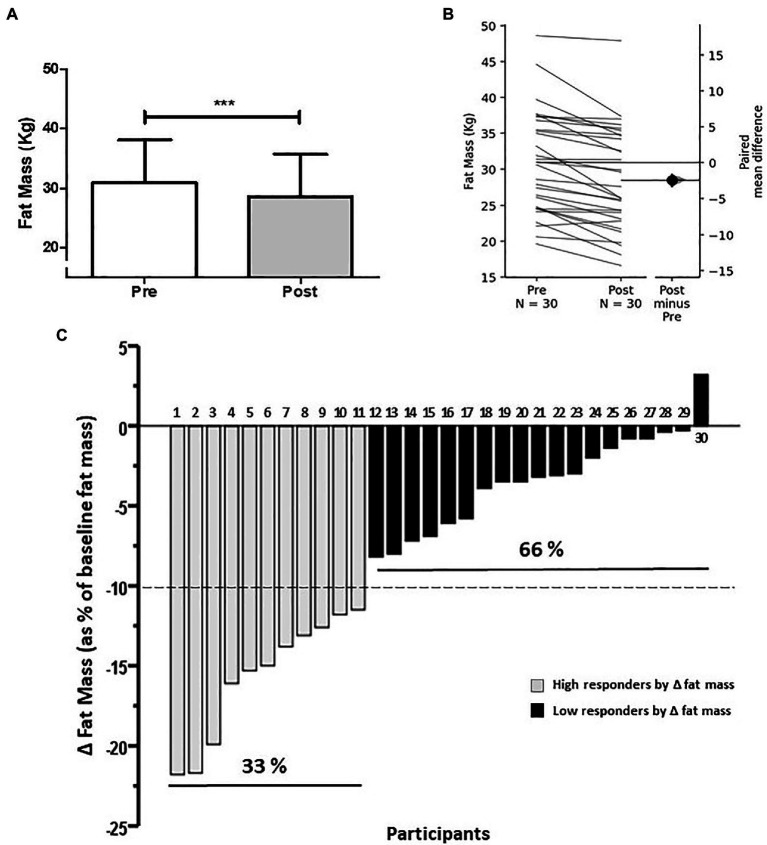
Average changes **(A)** and individual response **(B,C)** to decrease absolute fat mass after 12-week intervention (**A**: absolute fat mass; **B**: Gardner Altman Plot with individual responses on the left axes as a line graph and the mean difference between groups on floating axes on the right; and **C**: individual changes in delta fat mass as % of baseline absolute fat mass). Numbers on the bars in panel **(C)** represent the order of magnitude in the individual response to reduce fat mass. High and low responders are identified in relation to the ability to reduce fat mass. ^***^p < 0.0001.

At the group level, the intervention induced a reduction of absolute fat mass (i.e., kilograms) by 7.8% (30.9 ± 7.2 vs. 28.5 ± 7.2 kg; *p* < 0.0001; Figure 2A). The effect size of this group reduction in absolute fat mass was a mean difference of −2.4 kg (95%CI −3.23 to −1.76; Figure 2B). In addition, this group reduction in fat mass was also evidenced as a decrease in body fat percentage (fat mass divided by total body mass, multiplied by 100; 39.8 ± 4.3 vs. 37.8 ± 4.9%; *p* < 0.0001). On an individual level, wide inter-individual variability was found for absolute fat mass reduction (Figure 2C), allowing to separate individuals who lost ≥ 10% of initial absolute fat mass (i.e., kilograms) and individuals who lost < 10% of initial absolute fat mass, and classify them as HiRes or LoRes, respectively. The prevalence for HiRes was 33% (*n* = 11; Figure 2C, gray bars) and LoRes was 66% (*n* = 19; Figure 2C, black bars). The reduction in absolute fat mass in HiRes was 18.3% (Δ:−4.7 kg; 29.8 ± 8 vs. 25.2 ± 6.9 kg; *p* < 0.0001) and in LoRes was 3.6% (Δ:−1.1 kg; 31.6 ± 6.9 vs. 30.5 ± 6.7 kg; *p* < 0.0001), and the between-groups comparisons in the post-intervention values were statistically significant (*p* < 0.05). It is noteworthy that HiRes and LoRes groups were statistically similar in the demographic, physical, and physiological variables assessed at baseline (Table 1). Moreover, there was no difference in hypocaloric diet compliance between HiRes and LoRes (3.6 ± 0.5 vs. 3.3 ± 3.5 points; *p* = 0.3211). Numbers on top of each column in Figure 2C represent the identification number of each subject according to their order of magnitude in the individual response to reduce fat mass after the intervention. These numbers and the classification as HiRes or LoRes to reduce fat mass were maintained in the subsequent figures to identify the individual response of these same subjects in the other study variables. Furthermore, it is important to mention that the magnitude of the fat mass changes did not correlate with baseline body mass (*r* = −0.221, *p* = 0.242) or fat mass (*r* = −0.069, *p* = 0.716).

**Figure 3:**
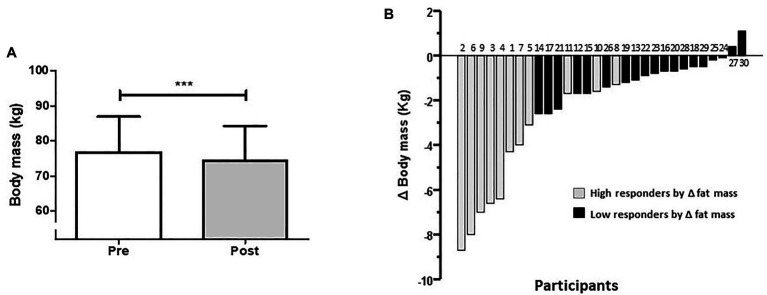
Average changes **(A)** and individual response **(B)** to decrease body mass after the 12-week intervention. Subjects with a high or low response to reduce their fat mass are represented with gray and black bars, respectively. Numbers on the bars **(B)** represent the order of magnitude in the individual response to reduce fat mass. High and low responders are identified in relation to the ability to reduce fat mass. ^***^p < 0.0001.

At the group level, there was a reduction in total body mass (76.7 ± 10.1 vs. 74.4 ± 9.9 kg; *p* < 0.0001; Figure 3A). The effect size of this group reduction in body mass was a mean difference of −2.36 kg (95%CI −3.43 to −1.61). Individual changes in body mass from highest to lowest response are presented in Figure 3B, with HiRes represented with gray bars and LoRes with black bars (the subject number is the same as Figure 2C). The reduction in body mass in HiRes was 6.3% (76.1 ± 11.1 vs. 71.3 ± 9.9 kg; *p* < 0.0001) and in LoRes was 1.3% (77.2 ± 9.8 vs. 76.2 ± 9.7 kg; *p* = 0.0004) but without statistically significant difference between groups observed in the post-intervention values. Moreover, there were no significant pre-post changes in FFM (45.9 ± 3.5 vs. 45.9 ± 3.5 kg; *p* = 0.8813).

**Figure 4:**
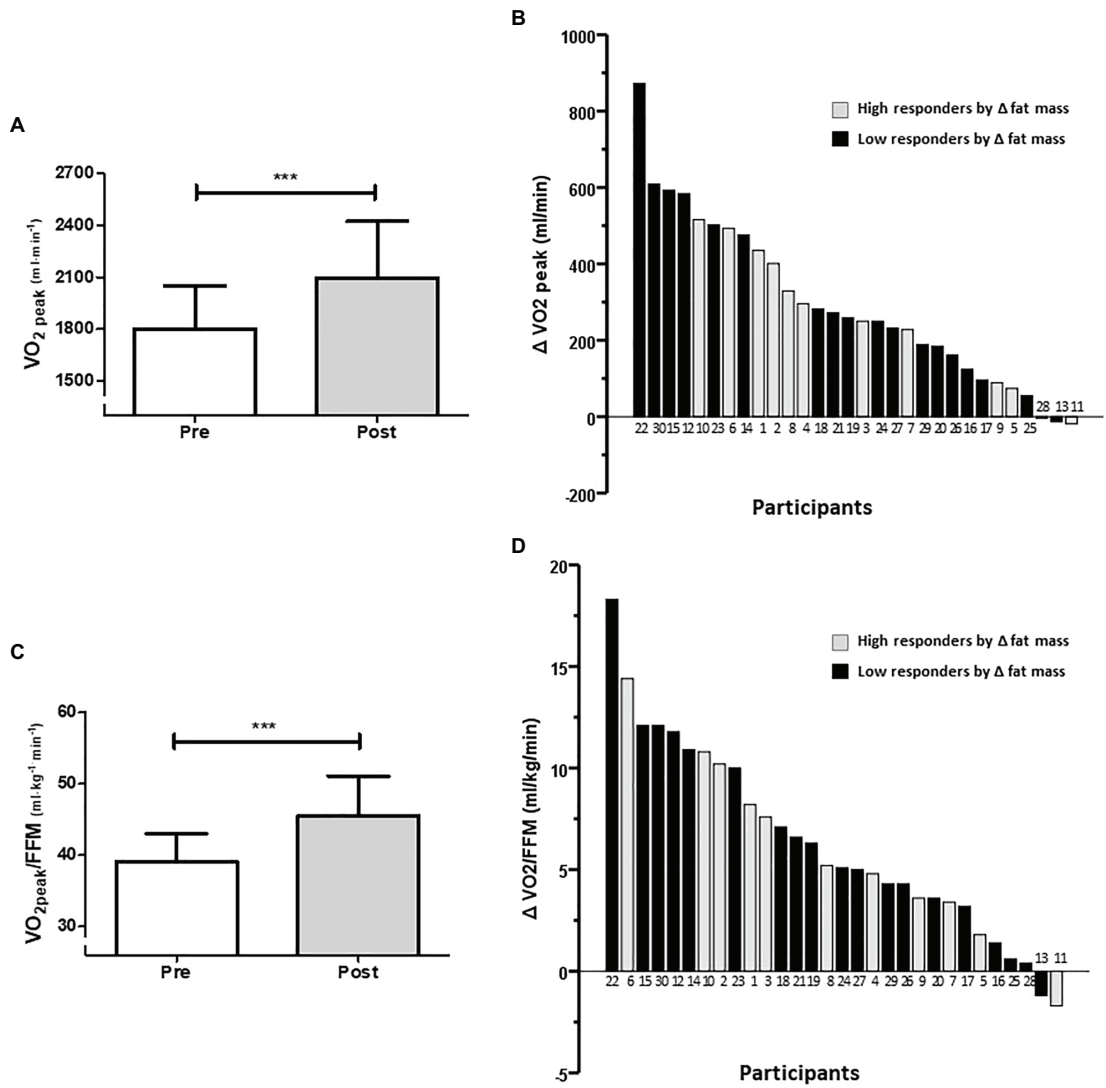
Average changes **(A,C)** and individual response **(B,D)** to increase cardiorespiratory fitness (CRF) after 12-week intervention (**A,B**: absolute VO_2_peak; **C,D**: VO_2_peak relative to fat-free mass). Numbers below the bars **(B,D)** represent the order of magnitude in the individual response to reduce fat mass. High and low responders are identified in relation to the ability to reduce fat mass. ^***^p < 0.0001.

Related to CRF, for the whole group, there was an increased absolute VO_2_peak by 14.0%. Paired *t*-tests revealed differences between baseline and after intervention measures for absolute VO_2_peak (1,800 ± 249 vs. 2,094 ± 328 ml/min; *p* < 0.0001; Figure 4A) and for VO_2_peak relative to FFM (VO_2_peak/FFM; 39.2 ± 3.9 vs. 45.5 ± 5.6 ml/kg/min; *p* < 0.0001; Figure 4C). The effect size of this group increment in absolute VO_2_peak was a mean difference of +294 ml/min (95%CI 213–374), and for VO_2_peak/FFM was +6.35 ml/kg/min (95%CI 4.57–8.14). Individual changes in absolute and relative VO_2_peak are presented in Figures 4B,D, respectively. VO_2_peak relative to total body mass was not used considering pre-post differences in total body mass. The increment in absolute VO_2_peak in HiRes was 14.9% (1,888 ± 266 vs. 2,169 ± 322 ml/min; *p* = 0.0004) and in LoRes was 17.2% (1,750 ± 230 vs. 2,051 ± 332 ml/min; *p* < 0.0001) but without statistically significant difference between groups observed in the post-intervention values. Furthermore, there were significant improvement in exercise performance, measured as increases in PPO by 19.8% (128.3 ± 17.0 vs. 160.0 ± 24.2 W; *p* < 0.0001), AT by 16.7% (58.0 ± 11.9 vs. 69.6 ± 14.2 W; *p* = 0.0025), and maximal ventilation (VEmax) by 14.1% (66.3 ± 15.2 vs. 77.2 ± 13.7 L/min; *p* = 0.0021). There was no pre-post change in maximal respiratory rate (RRmax; 36.9 ± 8.2 vs. 39.2 ± 5.5 breath/min; *p* = 0.05).

**Figure 5:**
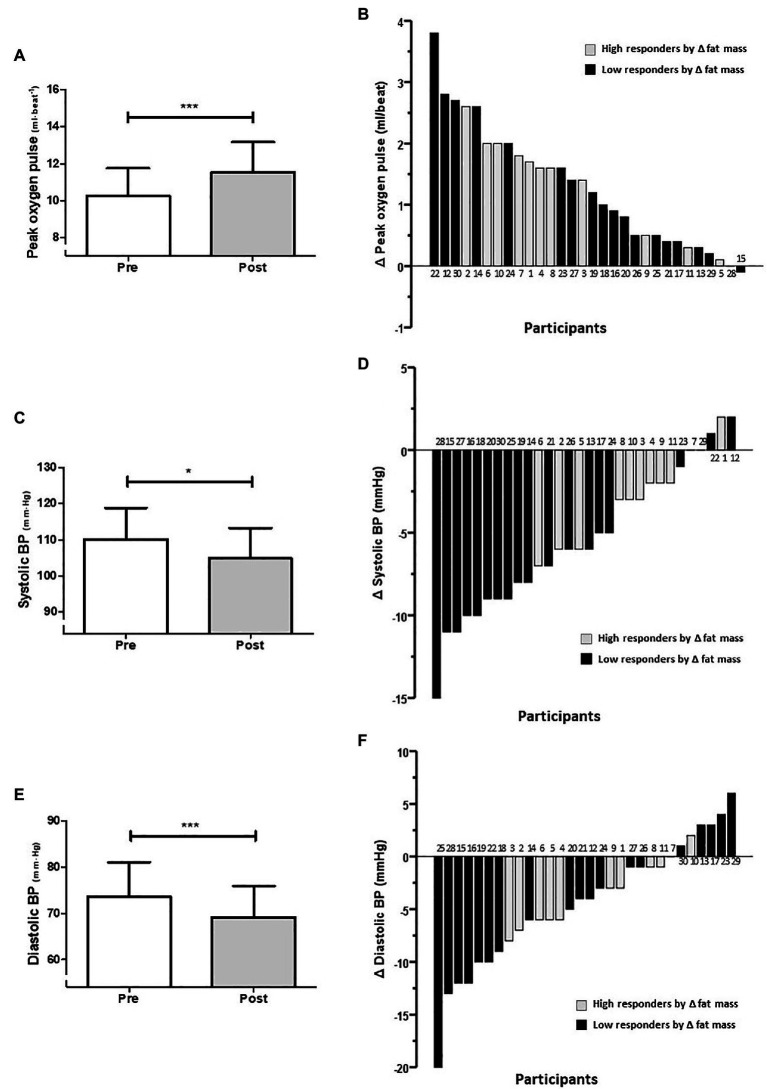
Average changes **(A,C,E)** and individual response **(B,D,F)** in cardiovascular outcomes after 12-week intervention [**A,B**: peak oxygen pulse; **C,D**: systolic blood pressure (SBP); and **E,F**: diastolic blood pressure (DBP)]. Numbers above and below the bars **(B,D,F)** represent the order of magnitude in the individual response to reduce fat mass. High and low responders are identified in relation to the ability to reduce fat mass. ^***^p < 0.0001; ^*^p < 0.05.

Within the cardiovascular adaptations to 12-week exercise intervention, a 10.4% increase was observed in the group average for O_2_ pulse (10.3 ± 1.5 vs. 11.5 ± 1.6 ml/beat; *p* < 0.0001; Figure 5A), which is a measure of maximum oxygen consumed per heartbeat. The effect size of this group increment in peak O_2_ pulse was a mean difference of +1.2 ml/beat (95%CI 0.91–1.65]. Individual changes in peak O_2_ pulse are presented in Figure 5B. In addition, decreases of 5.1 and 6.4% were observed for systolic blood pressure (SBP; 110.2 ± 8.7 vs. 104.9 ± 8.4 mmHg; *p* = 0.02; Figure 5C), and DBP (73.6 ± 7.5 vs. 69.2 ± 6.8 mmHg; *p* = 0.0003; Figure 5E), respectively. The effect size of this group reduction in SBP was a mean difference of −5.3 mmHg (95%CI −3.83 to −6.87), and for DBP was −4.4 mmHg (95%CI −2.57 to −6.63). Individual changes in SBP and DBP are presented in Figures 5D,F, respectively. The reduction in SBP in HiRes was 2.7% (108.9 ± 9.4 vs. 106.0 ± 9.8 mmHg; *p* = 0.357) and in LoRes was 5.6% (110.9 ± 8.4 vs. 104.7 ± 9.1 mmHg; *p* = 0.03) with a significant difference only in the LoRes group. When comparing reductions in DBP levels in the HiRes and LoRes groups, no differences were found.

## Discussion

We characterized the physiological effects and inter-individual variability on fat mass and other health-related and physical performance outcomes after 12 weeks of HIIT in overweight/obese adult women. The uniqueness of the present study was that it shows the inter-individual variability to HIIT in overweight/obese young adult women, classified as LoRes or HiRes for a particular variable, i.e., absolute fat mass reduction, in response to a specific supervised 12-week exercise program added to a slight dietary energy restriction. This intervention caused an improvement in multiple health-related and physical performance outcomes, i.e., reductions in absolute fat mass (Δ% = −7.8%, equivalent to −2.4 kg), body fat percentage (Δ% = −5.0%), total body mass (Δ% = −3.1%), SBP (Δ% = −5.1%), DBP (Δ% = −6.4%), and increases in absolute VO_2_peak (Δ% = +14.0%), relative VO_2_peak (Δ% = +13.8%), PPO (Δ% = +19.8%), anaerobic threshold (Δ% = +16.7%), maximal ventilation (Δ% = +14.1%), and O_2_pulse (Δ% = +10.4%). These results agree with several reviews and meta-analyses demonstrating that HIIT interventions allow multiple beneficial effects for health in a time-efficient manner, both in adults with obesity (Turk et al., 2017; Rugbeer et al., 2021) and lifestyle-induced cardiometabolic diseases (Gibala et al., 2012; Weston et al., 2014; Batacan et al., 2017; Maillard et al., 2018; Viana et al., 2019). Moreover, the meta-analysis by Dupuit et al. (2020) shows that cycling HIIT programs over 8 weeks are most effective in reducing fat and body mass in women before menopause (Dupuit et al., 2020). Correspondingly, the systematic review and meta-analysis by Johns et al. (2014) reported that programs combining exercise and diet are more effective in the short and long term for behavioral weight management (Johns et al., 2014). Also, the systematic review and meta-analysis by Clark (2015) reported that the combination of exercise and diet is more effective in producing changes in body composition, recommending interventions that generate large metabolic stress (induced by high levels of endurance exercise or resistance training) and not focused on energy imbalance in overweight adults (Clark, 2015). The reduction of fat mass after behavioral interventions is essential, as shown in the recent systematic review by Abe et al. (2021), where changes in total body fat have been associated with changes in visceral and subcutaneous adipose tissue following caloric restriction, caloric restriction plus exercise, and exercise alone interventions (Abe et al., 2021). However, beyond the good average group responses found in the present study, a wide range of responses was appreciated in each study variable individually.

Regarding the cut-off point used to classify subjects with a high or low response to the reduction of their absolute fat mass (kg), mention that there are different methods to make this differentiation (clinical cut-off points, within-subjects coefficient of variation, typical error of measurement, or two times the typical error) but without a consensus on which is the most appropriate method to differentiate these groups. The criterion used in this study was similar to previous studies, given an expected biological expression (Milagro et al., 2013; Parr et al., 2016). Parr et al. (2016) used similar cut-off points to evaluate circulating microRNA in the bloodstream (c-miR) before and after a 16-week intervention with exercise and diet in obese/overweight subjects. They observed that HiRes subjects (>10% reduction) have lower c-miR-935 expression pre- and post-intervention, knowing that c-miR-935 could be involved in modulating the expression of different metabolism-related genes (Parr et al., 2016). Similarly, Milagro et al. (2013) used a 5% cut-off point to assess differential expression of different miRNA in blood cells between HiRes and LoRes groups, but in a short-term (8 weeks) diet intervention without exercise training in excess body weight women (Milagro et al., 2013). Furthermore, the relevance of the cut-off point used in this study is supported by a systematic review comparing the effects of hypocaloric diet versus exercise (Verheggen et al., 2016), where the authors demonstrated that in the absence of weight loss, body fat reduction was much higher with exercise (6.1%) than diet (1.1%). Therefore, a 10% absolute fat mass reduction is a reasonably strict cut-off point to judge the failure/success of a 12-week intervention with exercise and diet.

Most of the previous studies reporting inter-individual variability have shown the prevalence of non-responders for a single outcome variable but without identifying the individual response of each subject for the primary variable and comparing it with the other study variables. For this study, the modification of fat mass was considered the primary outcome variable since it is a strong predictor of morbidity and mortality (Verheggen et al., 2016). Therefore, it was interesting for us to observe the individual response of each subject on other physical performance and health-related study variables, maintaining their classification as high-responder or low-responder for fat mass reduction and their corresponding identification number in the other study variables. Thus, subjects with more remarkable changes in fat mass (HiRes; Figure 2C, gray bars) were also those who had a more considerable reduction in total body mass (Figure 3B, gray bars); however, this was not the case for the rest of variables studied. Similar inter-individual variability to reduce fat mass has been observed in previous studies reporting the prevalence of non-responders after a HIIT intervention in subjects with cardiometabolic disorders (Álvarez et al., 2017b) and after moderate-intensity continuous training in overweight and obese subjects (Álvarez et al., 2012).

On the other hand, HIIT produces significant increases in CRF (Weston et al., 2014), which is highly relevant since improving VO_2_peak reduces the risk of all-cause mortality and cardiovascular events (Kodama et al., 2009). Nevertheless, we observe that improvement in CRF (i.e., VO_2_peak) after our 12-week intervention occurs with wide inter-individual variability, and this seems to occur independently of the high or low fat-mass response (Figure 4B, black bars). This pattern did not change when the VO_2_peak was normalized by FFM (Figure 4D, black bars). Since Bouchard et al. (1999) published their article, inter-individual variability of VO_2_peak in response to different exercise interventions has been one of the most widely reported topics (Bouchard et al., 1999). However, according to Ross et al. (2015), the frequency of non-responders decreases until it disappears; with increasing intensity and duration of a 24-week exercise program (Ross et al., 2015). Correspondingly, Montero and Lundby (2017) reported that CRF non-responders prevalence is abolished by increasing the dose of exercise in a 6-week endurance training program (Montero and Lundby, 2017).

Similarly, the individual responses for cardiovascular outcomes after the 12-week intervention showed a wide variability to increase the peak O_2_ pulse (Figure 5B). This improvement can be interpreted as a reflection of a greater stroke volume during maximum exercise (Mezzani, 2017), suggesting cardiac adaptations. Wide individual variability in reducing systolic and DBP (Figures 5D,F) were appreciated. It is worth mention that regardless that all participants in the present study were normotensive, they still showed improvements in their blood pressure levels, in agreement with a meta-analysis by Cornelissen and Smart (2013). They found that different exercise modalities effectively reduce blood pressure in normotensive and hypertensive subjects (Cornelissen and Smart, 2013). Thus, we could speculate that improvements were produced on endothelial function after the 12-week HIIT and diet intervention.

Among the numerous factors explaining the wide interindividual variability on exercise-induced fat and body mass loss, it can be cited: sex, ethnicity, muscle fiber type, body fat depot, diet, physical activity, sedentary behaviors, stress, sleep deprivation, adenovirus-36, and others (Boutcher and Dunn, 2009). Additionally, genetic and epigenetic factors could be playing a vital role in this exercise response heterogeneity (Sparks, 2017; Williamson et al., 2017; Hagstrom and Denham, 2018).

Our results corroborate the understanding that although there are high and low responders to exercise interventions, this classification is appropriate only for a single parameter and a specific exercise protocol. In this regard, Barbalho et al. (2017) study demonstrated that all participants in a 12-week resistance training showed improvements in at least one study outcome; therefore, there were no non-responders (Barbalho et al., 2017). This topic is genuinely relevant because it can motivate overweight and obese people to exercise, particularly when a time-efficient exercise strategy is used (Lidegaard et al., 2016). Thus, exercise modalities such as HIIT cause greater enjoyment than traditional programs of moderate-intensity continuous training (Thum et al., 2017) and in a time-efficient manner (Gillen and Gibala, 2014). Consequently, participants in these programs must be informed about the improvements in the different physical and health-related outcome variables during and after the interventions beyond reducing fat mass or body mass. This critical information will increase their adherence and motivation to behavioral changes to improve their health status.

The strengths of our study include the rigorous design of the exercise protocol monitored individually on a series-by-series and session-by-session basis during the 12-week program. Another strength was the individually prescribed home hypocaloric diet, as most similar studies acknowledge a lack of dietary control and only report that subjects were encouraged to maintain their usual diet during the exercise intervention period. On the other hand, one of its limitations was the lack of a no-intervention control group. Another limitation was using a BIA to measure body composition variables, as this is not considered the “gold standard” method.

In conclusion, a 12-week supervised HIIT program added to a slight dietary energy restriction effectively improved fat mass, body mass, blood pressure, and CRF. However, a wide range of inter-individual variability was observed in the adaptative response to the intervention. Furthermore, subjects classified as LoRes for fat mass reduction could be HiRes in many other health-related and physical performance outcomes. Thus, the beneficial effects of exercise in obese and overweight women go beyond the adaptive response to a single outcome variable such as fat mass or total body mass reduction.

## Data Availability Statement

The raw data supporting the conclusions of this article are available from the corresponding author upon reasonable request.

## Ethics Statement

The studies involving human participants were reviewed and approved by Scientific Ethics Committee at Universidad de La Frontera, Temuco, Chile. The patients/participants provided their written informed consent to participate in this study.

## Author Contributions

All authors have read the manuscript and agreed with the content. OA-M, ED, and LS conceived and designed the study. OA-M and NM-M performed the experiments. OA-M and ED analyzed the data. ED and LS contributed reagents, materials, and analysis tools and reviewed and edited the manuscript. OA-M wrote the paper. All authors contributed to the article and approved the submitted version.

## Conflict of Interest

The authors declare that the research was conducted in the absence of any commercial or financial relationships that could be construed as a potential conflict of interest.

## Publisher’s Note

All claims expressed in this article are solely those of the authors and do not necessarily represent those of their affiliated organizations, or those of the publisher, the editors and the reviewers. Any product that may be evaluated in this article, or claim that may be made by its manufacturer, is not guaranteed or endorsed by the publisher.

## References

[ref1] Abe T. Song J. S. Bell Z. W. Wong V. Spitz R. W. Yamada Y.. (2021). Comparisons of calorie restriction and structured exercise on reductions in visceral and abdominal subcutaneous adipose tissue: a systematic review. Eur. J. Clin. Nutr. 10.1038/s41430-021-00942-1 [Epub ahead of print] 34040197

[ref2] Álvarez C. Ramirez R. Flores M. Zuniga C. Celis-Morales C. A. (2012). Efectos del ejercicio fisico de Alta intensidad y sobrecarga en parametros de salud metabolica en mujeres sedentarias, pre-diabeticas con sobrepeso u obesidad. Rev. Med. Chil. 140, 1289–1296. 10.4067/S0034-98872012001000008, PMID: 23559286

[ref3] Álvarez C. Ramirez-Campillo R. Lucia A. Ramirez-Velez R. Izquierdo M. (2019). Concurrent exercise training on hyperglycemia and comorbidities associated: non-responders using clinical cutoff points. Scand. J. Med. Sci. Sports 29, 952–967. 10.1111/sms.13413, PMID: 30825342

[ref4] Álvarez C. Ramirez-Campillo R. Ramirez-Velez R. Izquierdo M. (2017a). Prevalence of non-responders for glucose control markers after 10 weeks of high-intensity interval training in adult women with higher and lower insulin resistance. Front. Physiol. 8:479. 10.3389/fphys.2017.00479, PMID: 28729841 PMC5498508

[ref5] Álvarez C. Ramírez-Campillo R. Ramírez-Vélez R. Izquierdo M. (2017b). Effects and prevalence of nonresponders after 12 weeks of high-intensity interval or resistance training in women with insulin resistance: a randomized trial. J. Appl. Physiol. 122, 985–996. 10.1152/japplphysiol.01037.2016, PMID: 28153946

[ref6] Andrade-Mayorga O. Mancilla R. Díaz E. Álvarez C. (2020). Heart rate during an exercise test and acute high-intensity interval training in type 2 diabetes. Int. J. Sports Med. 41, 365–372. 10.1055/a-1015-0591, PMID: 32045951

[ref7] Andreato L. V. Esteves J. V. Coimbra D. R. Moraes A. J. P. de Carvalho T. (2019). The influence of high-intensity interval training on anthropometric variables of adults with overweight or obesity: a systematic review and network meta-analysis. Obes. Rev. 20, 142–155. 10.1111/obr.12766, PMID: 30450794

[ref8] Astorino T. A. deRevere J. Anderson T. Kellogg E. Holstrom P. Ring S.. (2018). Change in VO_2_max and time trial performance in response to high-intensity interval training prescribed using ventilatory threshold. Eur. J. Appl. Physiol. 118, 1811–1820. 10.1007/s00421-018-3910-3, PMID: 29923111

[ref9] Astorino T. A. Schubert M. M. (2014). Individual responses to completion of short-term and chronic interval training: a retrospective study. PLoS One 9:e97638. 10.1371/journal.pone.0097638, PMID: 24847797 PMC4029621

[ref10] Barbalho M. S. M. Gentil P. Izquierdo M. Fisher J. Steele J. Raiol R. A. (2017). There are no no-responders to low or high resistance training volumes among older women. Exp. Gerontol. 99, 18–26. 10.1016/j.exger.2017.09.003, PMID: 28918362

[ref11] Batacan R. B. Duncan M. J. Dalbo V. J. Tucker P. S. Fenning A. S. (2017). Effects of high-intensity interval training on cardiometabolic health: a systematic review and meta-analysis of intervention studies. Br. J. Sports Med. 51, 494–503. 10.1136/bjsports-2015-095841, PMID: 27797726

[ref12] Bonafiglia J. T. Nelms M. W. Preobrazenski N. LeBlanc C. Robins L. Lu S.. (2018). Moving beyond threshold-based dichotomous classification to improve the accuracy in classifying non-responders. Phys. Rep. 6:e13928. 10.14814/phy2.13928, PMID: 30488594 PMC6429972

[ref13] Bonafiglia J. T. Rotundo M. P. Whittall J. P. Scribbans T. D. Graham R. B. Gurd B. J. (2016). Inter-individual variability in the adaptive responses to endurance and sprint interval training: a randomized crossover study. PLoS One 11:e0167790. 10.1371/journal.pone.0167790, PMID: 27936084 PMC5147982

[ref14] Booth F. W. Roberts C. K. Laye M. J. (2012). Lack of exercise is a major cause of chronic diseases. Compr. Physiol. 2, 1143–1211. 10.1002/cphy.c110025, PMID: 23798298 PMC4241367

[ref15] Bouchard C. An P. Rice T. Skinner J. S. Wilmore J. H. Gagnon J.. (1999). Familial aggregation of VO(2max) response to exercise training: results from the HERITAGE family study. J. Appl. Physiol. 87, 1003–1008. 10.1152/jappl.1999.87.3.1003, PMID: 10484570

[ref16] Bouchard C. Blair S. N. Church T. S. Earnest C. P. Hagberg J. M. Hakkinen K.. (2012). Adverse metabolic response to regular exercise: is it a rare or common occurrence? PLoS One 7:e37887. 10.1371/journal.pone.0037887, PMID: 22666405 PMC3364277

[ref17] Bouchard C. Rankinen T. (2001). Individual differences in response to regular physical activity. Med. Sci. Sports Exerc. 33, S446–S451. 10.1097/00005768-200106001-00013, PMID: 11427769

[ref18] Boutcher S. H. Dunn S. L. (2009). Factors that may impede the weight loss response to exercise-based interventions. Obes. Rev. 10, 671–680. 10.1111/j.1467-789X.2009.00621.x, PMID: 19538438

[ref19] Cancello R. Soranna D. Brunani A. Scacchi M. Tagliaferri A. Mai S.. (2018). Analysis of predictive equations for estimating resting energy expenditure in a large cohort of morbidly obese patients. Front. Endocrinol. 9:367. 10.3389/fendo.2018.00367, PMID: 30090085 PMC6068274

[ref20] Cheneviere X. Borrani F. Sangsue D. Gojanovic B. Malatesta D. (2011). Gender differences in whole-body fat oxidation kinetics during exercise. Appl. Physiol. Nutr. Metab. 36, 88–95. 10.1139/H10-086, PMID: 21326382

[ref21] Chrzanowski-Smith O. J. Piatrikova E. Betts J. A. Williams S. Gonzalez J. T. (2019). Variability in exercise physiology: can capturing intra-individual variation help better understand true inter-individual responses? Eur. J. Sport Sci. 20, 1–9. 10.1080/17461391.2019.1655100 31397212

[ref22] Clark J. E. (2015). Diet, exercise or diet with exercise: comparing the effectiveness of treatment options for weight-loss and changes in fitness for adults (18-65 years old) who are overfat, or obese; systematic review and meta-analysis. J. Diabetes Metab. Disord. 14:31. 10.1186/s40200-015-0154-1, PMID: 25973403 PMC4429709

[ref23] Cornelissen V. A. Smart N. A. (2013). Exercise training for blood pressure: a systematic review and meta-analysis. J. Am. Heart Assoc. 2:e004473. 10.1161/JAHA.112.004473, PMID: 23525435 PMC3603230

[ref24] de Lannoy L. Clarke J. Stotz P. J. Ross R. (2017). Effects of intensity and amount of exercise on measures of insulin and glucose: analysis of inter-individual variability. PLoS One 12:e0177095. 10.1371/journal.pone.0177095, PMID: 28493912 PMC5426643

[ref25] Dupuit M. Maillard F. Pereira B. Marquezi M. L. Lancha A. H. Jr. Boisseau N. (2020). Effect of high intensity interval training on body composition in women before and after menopause: a meta-analysis. Exp. Physiol. 105, 1470–1490. 10.1113/EP088654, PMID: 32613697

[ref26] FAO/WHO/UNU (2004). Human energy requirements: report of a joint FAO/WHO/UNU Expert Consultation, Rome 17–24 October 2001. Rome: Food and Agriculture Organization of the United Nations.

[ref27] Gibala M. J. Little J. P. Macdonald M. J. Hawley J. A. (2012). Physiological adaptations to low-volume, high-intensity interval training in health and disease. J. Physiol. 590, 1077–1084. 10.1113/jphysiol.2011.224725, PMID: 22289907 PMC3381816

[ref28] Gillen J. B. Gibala M. J. (2014). Is high-intensity interval training a time-efficient exercise strategy to improve health and fitness? Appl. Physiol. Nutr. Metab. 39, 409–412. 10.1139/apnm-2013-0187, PMID: 24552392

[ref29] Gurd B. J. Giles M. D. Bonafiglia J. T. Raleigh J. P. Boyd J. C. Ma J. K.. (2016). Incidence of nonresponse and individual patterns of response following sprint interval training. Appl. Physiol. Nutr. Metab. 41, 229–234. 10.1139/apnm-2015-0449, PMID: 26854820

[ref30] Hagstrom A. D. Denham J. (2018). microRNAs in high and low responders to resistance training in breast cancer survivors. Int. J. Sports Med. 39, 482–489. 10.1055/a-0592-7691, PMID: 29698983

[ref31] Hepler C. Gupta R. K. (2017). The expanding problem of adipose depot remodeling and postnatal adipocyte progenitor recruitment. Mol. Cell. Endocrinol. 445, 95–108. 10.1016/j.mce.2016.10.011, PMID: 27743993 PMC5346481

[ref32] Heymsfield S. B. Wadden T. A. (2017). Mechanisms, pathophysiology, and management of obesity. N. Engl. J. Med. 376:1492. 10.1056/NEJMc1701944, PMID: 28402780

[ref33] Ho J. Tumkaya T. Aryal S. Choi H. Claridge-Chang A. (2019). Moving beyond P values: data analysis with estimation graphics. Nat. Methods 16, 565–566. 10.1038/s41592-019-0470-3, PMID: 31217592

[ref34] Jebb S. A. Cole T. J. Doman D. Murgatroyd P. R. Prentice A. M. (2000). Evaluation of the novel Tanita body-fat analyser to measure body composition by comparison with a four-compartment model. Br. J. Nutr. 83, 115–122. 10.1017/S0007114500000155, PMID: 10743490

[ref35] Johns D. J. Hartmann-Boyce J. Jebb S. A. Aveyard P. (2014). Diet or exercise interventions vs combined behavioral weight management programs: a systematic review and meta-analysis of direct comparisons. J. Acad. Nutr. Diet. 114, 1557–1568. 10.1016/j.jand.2014.07.005, PMID: 25257365 PMC4180002

[ref36] King N. A. Hopkins M. Caudwell P. Stubbs R. J. Blundell J. E. (2008). Individual variability following 12 weeks of supervised exercise: identification and characterization of compensation for exercise-induced weight loss. Int. J. Obes. 32, 177–184. 10.1038/sj.ijo.0803712, PMID: 17848941

[ref37] Kodama S. Saito K. Tanaka S. Maki M. Yachi Y. Asumi M.. (2009). Cardiorespiratory fitness as a quantitative predictor of all-cause mortality and cardiovascular events in healthy men and women: a meta-analysis. JAMA 301, 2024–2035. 10.1001/jama.2009.681, PMID: 19454641

[ref38] Levine J. A. (2005). Measurement of energy expenditure. Public Health Nutr. 8, 1123–1132. 10.1079/PHN2005800, PMID: 16277824

[ref39] Lidegaard L. P. Schwennesen N. Willaing I. Faerch K. (2016). Barriers to and motivators for physical activity among people with type 2 diabetes: patients' perspectives. Diabet. Med. 33, 1677–1685. 10.1111/dme.13167, PMID: 27279343

[ref40] Maillard F. Pereira B. Boisseau N. (2018). Effect of high-intensity interval training on total, abdominal and visceral fat mass: a meta-analysis. Sports Med. 48, 269–288. 10.1007/s40279-017-0807-y, PMID: 29127602

[ref41] Mann T. N. Lamberts R. P. Lambert M. I. (2014). High responders and low responders: factors associated with individual variation in response to standardized training. Sports Med. 44, 1113–1124. 10.1007/s40279-014-0197-3, PMID: 24807838

[ref42] Meule A. Papies E. K. Kübler A. (2012). Differentiating between successful and unsuccessful dieters validity and reliability of the perceived self-regulatory success in dieting scale. Appetite 58, 822–826. 10.1016/j.appet.2012.01.028, PMID: 22329988

[ref43] Mezzani A. (2017). Cardiopulmonary exercise testing: basics of methodology and measurements. Ann. Am. Thorac. Soc. 14, S3–S11. 10.1513/AnnalsATS.201612-997FR, PMID: 28510504

[ref44] Milagro F. I. Miranda J. Portillo M. P. Fernandez-Quintela A. Campion J. Martinez J. A. (2013). High-throughput sequencing of microRNAs in peripheral blood mononuclear cells: identification of potential weight loss biomarkers. PLoS One 8:e54319. 10.1371/journal.pone.0054319, PMID: 23335998 PMC3545952

[ref45] Montero D. Lundby C. (2017). Refuting the myth of non-response to exercise training: 'non-responders' do respond to higher dose of training. J. Physiol. 595, 3377–3387. 10.1113/JP273480, PMID: 28133739 PMC5451738

[ref46] NCD-RisC (2016). Trends in adult body-mass index in 200 countries from 1975 to 2014: a pooled analysis of 1698 population-based measurement studies with 19.2 million participants. Lancet 387, 1377–1396. 10.1016/S0140-6736(16)30054-X, PMID: 27115820 PMC7615134

[ref47] Ng M. Fleming T. Robinson M. Thomson B. Graetz N. Margono C.. (2014). Global, regional, and national prevalence of overweight and obesity in children and adults during 1980-2013: a systematic analysis for the global burden of disease study 2013. Lancet 384, 766–781. 10.1016/S0140-6736(14)60460-8, PMID: 24880830 PMC4624264

[ref48] Parr E. B. Camera D. M. Burke L. M. Phillips S. M. Coffey V. G. Hawley J. A. (2016). Circulating microrna responses between 'high' and 'low' responders to a 16-wk diet and exercise weight loss intervention. PLoS One 11:e0152545. 10.1371/journal.pone.0152545, PMID: 27101373 PMC4839646

[ref49] Petridou A. Siopi A. Mougios V. (2019). Exercise in the management of obesity. Metabolism 92, 163–169. 10.1016/j.metabol.2018.10.009, PMID: 30385379

[ref50] Raleigh J. P. Giles M. D. Scribbans T. D. Edgett B. A. Sawula L. J. Bonafiglia J. T.. (2016). The impact of work-matched interval training on VO2peak and VO2 kinetics: diminishing returns with increasing intensity. Appl. Physiol. Nutr. Metab. 41, 706–713. 10.1139/apnm-2015-0614, PMID: 27337599

[ref51] Ross R. de Lannoy L. Stotz P. J. (2015). Separate effects of intensity and amount of exercise on interindividual cardiorespiratory fitness response. Mayo Clin. Proc. 90, 1506–1514. 10.1016/j.mayocp.2015.07.024, PMID: 26455890

[ref52] Ross R. Goodpaster B. H. Koch L. G. Sarzynski M. A. Kohrt W. M. Johannsen N. M.. (2019). Precision exercise medicine: understanding exercise response variability. Br. J. Sports Med. 53, 1141–1153. 10.1136/bjsports-2018-100328, PMID: 30862704 PMC6818669

[ref53] Rugbeer N. Constantinou D. Torres G. (2021). Comparison of high-intensity training versus moderate-intensity continuous training on cardiorespiratory fitness and body fat percentage in persons with overweight or obesity: a systematic review and meta-analysis of randomized controlled trials. J. Phys. Act. Health 18, 610–623. 10.1123/jpah.2020-0335, PMID: 33837165

[ref54] Salvador Castell G. Serra-Majem L. Ribas-Barba L. (2015). What and how much do we eat? 24-hour dietary recall method. Nutr. Hosp. 31, 46–48. 10.3305/nh.2015.31.sup3.8750, PMID: 25719770

[ref55] Scharhag-Rosenberger F. Walitzek S. Kindermann W. Meyer T. (2012). Differences in adaptations to 1 year of aerobic endurance training: individual patterns of nonresponse. Scand. J. Med. Sci. Sports 22, 113–118. 10.1111/j.1600-0838.2010.01139.x, PMID: 20561283

[ref56] Sparks L. M. (2017). Exercise training response heterogeneity: physiological and molecular insights. Diabetologia 60, 2329–2336. 10.1007/s00125-017-4461-6, PMID: 29032385

[ref57] Thum J. S. Parsons G. Whittle T. Astorino T. A. (2017). High-intensity interval training elicits higher enjoyment than moderate intensity continuous exercise. PLoS One 12:e0166299. 10.1371/journal.pone.0166299, PMID: 28076352 PMC5226715

[ref58] Turk Y. Theel W. Kasteleyn M. J. Franssen F. M. E. Hiemstra P. S. Rudolphus A.. (2017). High intensity training in obesity: a meta-analysis. Obes. Sci. Pract. 3, 258–271. 10.1002/osp4.109, PMID: 29071102 PMC5598019

[ref59] Vazquez-Carrera M. (2016). Unraveling the effects of PPARbeta/delta on insulin resistance and cardiovascular disease. Trends Endocrinol. Metab. 27, 319–334. 10.1016/j.tem.2016.02.008, PMID: 27005447

[ref60] Verheggen R. J. Maessen M. F. Green D. J. Hermus A. R. Hopman M. T. Thijssen D. H. (2016). A systematic review and meta-analysis on the effects of exercise training versus hypocaloric diet: distinct effects on body weight and visceral adipose tissue. Obes. Rev. 17, 664–690. 10.1111/obr.12406, PMID: 27213481

[ref61] Viana R. B. Naves J. P. A. Coswig V. S. de Lira C. A. B. Steele J. Fisher J. P.. (2019). Is interval training the magic bullet for fat loss? A systematic review and meta-analysis comparing moderate-intensity continuous training with high-intensity interval training (HIIT). Br. J. Sports Med. 53, 655–664. 10.1136/bjsports-2018-099928, PMID: 30765340

[ref62] Weston K. S. Wisloff U. Coombes J. S. (2014). High-intensity interval training in patients with lifestyle-induced cardiometabolic disease: a systematic review and meta-analysis. Br. J. Sports Med. 48, 1227–1234. 10.1136/bjsports-2013-092576, PMID: 24144531

[ref63] Williamson P. J. Atkinson G. Batterham A. M. (2017). Inter-individual responses of maximal oxygen uptake to exercise training: a critical review. Sports Med. 47, 1501–1513. 10.1007/s40279-017-0680-8, PMID: 28097487

